# Are Aggregated Electronic Health Record Datasets Good for Research?

**DOI:** 10.1007/s11606-025-09808-9

**Published:** 2025-08-12

**Authors:** Neal D. Goldstein, Brianne Olivieri-Mui, Igor Burstyn

**Affiliations:** 1 Department of Epidemiology and Biostatistics, Dornsife School of Public Health, Drexel University, Philadelphia, USA; 2 Department of Microbiology and Immunology, College of Medicine, Drexel University, Philadelphia, PA USA; 3 Department of Public Health and Health Sciences, Bouve College of Health Sciences, Northeastern University, Boston, MA USA; 4 The Marcus Institute for Aging Research, Hebrew SeniorLife, Boston, MA USA; 5 Department of Environmental and Occupational Health, Dornsife School of Public Health, Drexel University, Philadelphia, PA USA

**Keywords:** electronic health records, data aggregation, validity, quantitative bias analysis

## Abstract

There has been a proliferation of large-scale electronic health record (EHR) data platforms that pool across multiple healthcare organizations, such as the National Institutes of Health’s *All of Us* in the federal space and TriNetX and Epic Cosmos in the commercial space. There are unique issues that occur when EHR data are aggregated across disparate healthcare systems beyond the general—and more well known—concerns about secondary analysis of EHR data from a single entity. In this article, we define aggregated EHR data, contrasting it to other real-world data sources, highlight benefits and challenges when working with aggregated EHR data, offer several “good practices” to address these challenges, and conclude by discussing whether it is appropriate to pool these data together or not.

*“If you’ve seen one EHR, you’ve seen one EHR.” *^1^

## INTRODUCTION

The use of electronic health record (EHR) data in biomedical research has increased proportional to the adoption of this technology in the healthcare setting even though these data were not created for research purposes. The challenges in using EHR data for research have been well described in the literature.^2,3^ A sequalae of these challenges is a threat to the validity of EHR-based research. Nevertheless, there is a potential advantage to the aggregation of multiple healthcare organizations’ EHR data on a large scale in understanding patterns and risk factors for disease provided the limitations of the validity and comparability of the data are understood.

**Figure 1:**
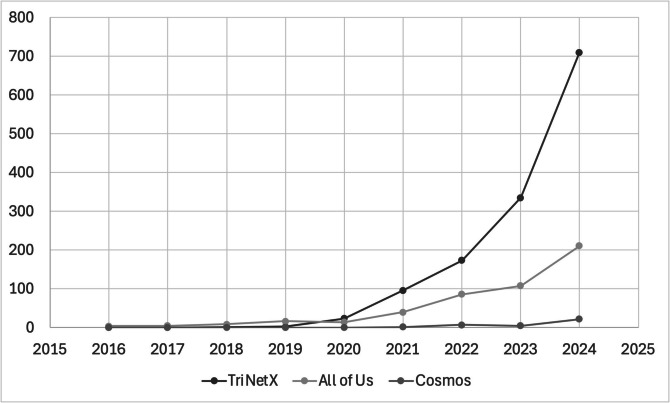
PubMed indexed citations by year that mention *All of Us*, TriNetX, or Epic Cosmos in the article title or abstract.

Interested parties seeking to amass a large-scale collection of real-world EHR data for research purposes include government-backed initiatives such as the National Institutes of Health’s *All of Us* research program, third-party companies such as TriNetX (TriNetX, LLC, Cambridge, MA), and EHR vendors such as Epic’s Cosmos (Epic Systems Corporation, Verona, WI). Figure 1 plots PubMed indexed citations from *All of Us*, TriNetX, and Cosmos, demonstrating the proliferation of their data in research publications. Though these resources are new, they have experienced an exponential increase in use and are one of an increasing number of platforms available to researchers that aggregate diverse hospital systems’ data. It therefore behooves researchers working with aggregated EHR data to be cognizant of the data limitations, while ensuring its prudent use. In this article, we define aggregated EHR data contrasting it to other real-world data sources, highlight benefits and challenges when working with aggregated EHR data, offer several “good practices” to address these challenges, and conclude by discussing whether it is appropriate to pool these data together or not with analogy from meta-analysis.

It is important to note at the outset that we are not commenting on the technical feasibility of aggregation; common data models (CDMs) have been widely used to standardize data capture across disparate healthcare standards and EHR vendor technology.^4^ We discuss implications of CDMs as they relate to data provenance later. We are also not criticizing any data provider or resource, and the included examples are merely for illustrative purposes.

## WHAT ARE AGGREGATED EHR DATA?

Our use of the term aggregated EHR data is meant to convey data that arise from *multiple* EHRs across *multiple* healthcare systems. The aggregation may be cross-sectional, occurring at a single time point (e.g., upon a healthcare system or patient enrollment with the aggregated platform), or longitudinal capturing multiple patient encounters over time. While the healthcare environment in the USA has continued to consolidate,^5^ and thus a single healthcare system’s EHR can include vast geographies and large patient populations, to be considered an aggregate, the data must pool across disparate EHRs.

There are other aggregated “real world” datasets used in clinical and epidemiological research (see Table 1 for comparisons). For example, all-payer claims databases pool insurance claims across multiple healthcare systems, but data generated from the healthcare billing process, as opposed to data generated from the care delivery process, contain different data elements and serve different research purposes. Nevertheless the challenges that we described herein are applicable to both data sources. Additionally, there may be some overlap between the challenges we describe from aggregated EHR data compared to institutional EHR data; in these cases, pooling across healthcare systems intensifies the ongoing challenge of working with these data for research purposes.

**Table 1:** Contrasting Single-Institution Electronic Health Record (EHR) Data, Aggregated EHR Data, and Insurance Claims Data for Clinical and Epidemiological Research

*Consideration*	**Institutional EHR**	**Aggregated EHR**	**Claims databases**
*Primary intention*	Patient care	Research	Billing and reimbursement
*Representativeness*	Patients who have accessed healthcare	Patients who have accessed healthcare at organizations enrolled in the platforms	Patients who make insurance claims
*Analytic unit*	Patient-encounter	Patient-encounter	Patient-claim
*Geography*	Narrow	Broad	Broad
*Data granularity*	High	Medium	Low
*Data quality*	As good as source	As good as extraction, transformation, and loading pipeline	As good as source
*Data strengths*	Biomedical data, clinical documentation	Diagnoses, medications, procedures	Diagnoses, medications, procedures
*Unstructured data*	Common	Uncommon	Uncommon
*Linkage to other sources*	By investigator or organization	By platform	By investigator or organization
*Protected health information*	Accessible	Not accessible	Not accessible

## BENEFITS OF AGGREGATED EHR DATA

There is widespread enthusiasm for aggregated EHR data. Researchers have extolled how a longitudinal collection of such data in *All of Us* “across different populations offers significant potential for understanding complex diseases, uncovering unknown relationships, and enhancing diagnostic and treatment options over the life course”.^6^ Aggregated EHR data may be useful for rare disease or adverse event detection as well as real-time surveillance on a broad geography, as was done during the COVID-19 pandemic.^4^ In contrast, a typical research paradigm for single-institution EHR data often necessitate a retrospective data retrieval, thus data lag analysis.

Another feature of aggregated EHR data is their relative ease of use. Our three examples—*All of Us*, TriNetX, and Cosmos—are accessible to researchers via online web-based platforms. The data have already been extracted from the source EHRs, transformed into a CDM, and loaded into each platform. Data dictionaries tend to be more commonly available with aggregated platforms (versus institutional EHRs) that fully describe data elements—e.g., the *All of Us* dictionaries are available here^7^—although not all elements may be consistently populated. End users can construct cohorts, perform exploratory analysis, and generate descriptive statistics within these platforms often by simple point-and-click interfaces. Whereas TriNetX and Cosmos integrate such analytic tools directly into their platforms without the need for statistical programming expertise, *All of Us* relies on third-party statistical software. Depending on data access and licensing agreements, patient-level datasets may also be obtained and analyzed.

A third benefit occurs when the aggregated data purveyor links the EHR to other sources, which may eliminate the need for manual linkages and third-party data sharing agreements. For example, *All of Us* combines EHR data with other supplementary data not routinely collected in a typical healthcare encounter including primary collection of social, behavioral, and lifestyle characteristics from surveys; genomic variants; and wearable devices.^8^ TriNetX offers insurance claims and mortality-linked datasets,^9^ overcoming the challenge of identifying medication use and death outside of the source EHR.

## CHALLENGES OF AGGREGATED EHR DATA

### Loss of Information

Patient-level data reported from EHRs to platforms such as TriNetX and Epic Cosmos in the USA are covered under the Health Insurance Portability and Accountability Act (HIPAA). To reuse these data for research purposes without consent of the patient requires de-identification or pseudonymization, which may remove or obscure useful information for researchers such as patient address (for spatial analysis) or exact dates of services (for longitudinal analysis). Date shifting may occur where relative times between events are preserved while exact dates are masked.^3^ By contrast in *All of Us*, the patient has been consented but access to certain additional data is controlled by tiers of data access.^10^ The public and registered tiers obscure or limit EHR data while the controlled tier provides the most complete picture.

Common to all data models is the requirement for some degree of harmonization through data extraction, transformation, and loading (ETL) pipelines. CDMs provide the table shells (what data could be captured), EHRs provide the data, and the transformation process populates the table shells in two steps. The first step changes the EHR data from the codes used by the host organization (e.g., International Classification of Diseases [ICD] codes) to the codes selected for harmonization (e.g., Systematized Nomenclature of Medicine [SNOMED] codes). The harmonized data are then populated into the table shells. This process incurs limitations in what granularity of data can be retained because, for example, there is no 1:1 conversion for ICD to SNOMED code such as are used in *All of Us*. Therefore, the accuracy of various data points can vary based on the transformation process and how well the harmonized coding represents the EHR data. This can be particularly challenging for health services research when data such as place of service, provider details, and even inpatient versus outpatient identifiers may not be available or accurate, despite being apparent and accessible in an institutional EHR research setting.

When data are accessed from an aggregated platform, the researcher may not have the opportunity to collect additional data from the EHR nor validate the data abstracted from the source EHR. An example of this would be medication adherence. While the source EHR contains an order for a medication, the accuracy of this order can vary, and it may be missing important information such as strength or duration of use.^11^ Moreover, whether the patient fulfilled this order and took the medicine as indicated may need to be determined via additional sources, such as linked insurance claims data^12^ or a patient self-report captured in providers’ notes.^13^ Unless data validation and linkage was done a priori or can be done at the researcher’s request, the utility of discrete medication data on an aggregated platform will be limited.

Unstructured EHR data from clinicians’ encounter and progress notes provide rich information on the context of the patient and healthcare process. These notes often include epidemiological determinants, such as housing or food stability, not historically available as structured data,^14^ but this also depends on the source EHR as structured data fields for such social determinants have become more common.^15^ These unstructured data are less likely to be available in aggregated data given the difficulty in anonymizing it. For example, unstructured data are not available to researchers in the present release of *All of Us*.^10^ Natural language processing (NLP) may have occurred prior to presentation of the data on the aggregated platform, such as to operationalize measures of the social determinants of health; however, the validity of these approaches will not be unknown to the end user without disclosure of an accompanying validation study.^3^ Further, the validation study itself may need to be repeated every time an EHR abstraction occurs as new or additional unstructured data are processed. Returning to the medication example, should lack of adherence be documented as a free text note, it may be unavailable to the researcher when using an aggregated EHR dataset. The consequence of this is the possibility of misclassification—assuming the patient had been adherent when they were not—as detailed below.

### Masking Heterogeneity and Nonindependence

Heterogeneity of effects occurs when associations of interest are importantly different in subgroups of patients. It is axiomatic in statistics that associations in a multi-factor problem should only be interpreted if one is certain that the associations of an independent and dependent variable do not differ depending on the presence of a third factor, known as an interaction among statisticians and an effect modifier among epidemiologists. In the case of EHR data pooled across diverse regions and healthcare systems, it seems reasonable to inquire whether pooling of effect estimates across such factors is justified. While there are many practical and scientific challenges to evaluating such heterogeneity of effects, causal claims are weakened when it is not possible to empirically examine perhaps the most important sources of effect heterogeneity: origin of the data from a distinct data source, exemplified by multiple EHRs contributing data.

Spatial analysis of healthcare data has demonstrated heterogeneity of healthcare utilization and outcomes such as asthma, obesity, infection, cancer, and heart disease, to name but a few.^16^ This heterogeneity has been observed across many geographic levels, from macro-level analyses by country to micro-level analyses by city.^17^ To undertake such analyses requires access to the patients’ addresses in the EHR. As mentioned earlier, patient address cannot be reported in aggregated datasets due to HIPAA privacy provisions. Even the ability to identify the specific healthcare system or EHR that is providing data may not be possible in such data sources. For example, TriNetX privacy practices state that the identification “of which [HIPAA] covered entity contributed which specific information about a patient” is not possible.^18^ TriNetX limits geographic identification to US Census Divisions (i.e., New England, Mid-Atlantic, South Atlantic, East South Central, West South Central, East North Central, West North Central, Mountain, Pacific). *All of Us* provides the first three digits of a ZIP code (this would be equivalent to the entire city of Philadelphia, PA, for example) but only in the highest tier of data access.^10^ As with TriNetX, *All of Us* does not allow identification of the source healthcare system. Consequently, important heterogeneity may be lost when performing an aggregated analysis. This may also be true in an institutional EHR setting where the healthcare system encompasses broad geographies and disparate markets, such as may occur when healthcare systems consolidate.^5^

Relatedly, patients within a given EHR may be more similar than patients aggregated across multiple EHRs. For example, the reputation of a hospital is a key factor in patient choice,^19^ so a statistical analysis of patients across many hospitals with varying reputations may violate the independence of observations assumption unless clustering by hospital can be modeled. Moreover, the type of healthcare provided, for example a cancer center, might contribute data specific to a subgroup of patients, while the primary care hospitals for the same patients may not contribute to the aggregation effort. The lack of understanding around type of facility (e.g., specialty care vs. primary care) and choice to use that facility not only obfuscates heterogeneity and the potential nonindependence of observations but poses significant threats to both internal and external validity discussed next.

### Exacerbation of Threats to Validity

There are several mechanisms by which EHR data, both institutional and aggregate, may compromise study validity.^2^ These can be broadly summarized as issues of nonrandom patient selection into healthcare and data accuracy and availability. First, lack of the representativeness of patients in healthcare systems can yield issues of selection bias, if healthcare seeking behavior is conditioned on the exposure and outcome under study, or lack of generalizability if there are unknown effect modifiers in the source population. Second, the accuracy and availability of data may result in information bias when measurement error or misclassification occurs and residual confounding if important covariates are not available or poorly represented by the aggregated platform’s ETL pipeline. Third, missing data, both at a patient level (e.g., missing lab value) and a visit level (e.g., patient received care at a healthcare facility that does not contribute to the data source), may result in information bias, selection bias, and confounding.

When these data are shared and aggregated together, the threats to validity are likely exacerbated. Suppose the accuracy of EHR data vary by institution, a not unreasonable assertion as variability in documentation has been observed within a single hospital.^20^ This presents two obstacles to the researcher. First, the number of variables that are subject to misclassification may increase. For example, researchers have compared the accuracy of ICD codes in the EHR for sepsis derived from validation studies conducted by various healthcare systems and noted wide variation in codes used and corresponding measures of accuracy.^21^ Under one sepsis definition, sensitivity ranged from 15 to 85% while specificity ranged from 70 to 100%. Relatedly, by design ETL operates on a subset of the EHR; therefore, the CDM table shells may have informative missingness dependent on the EHR source and quality. Continuing with the sepsis example, this could occur if the individual sepsis criteria were documented differently across the source EHRs and thereby inconsistently populated in the aggregated CDM. Second, the ability to bias-adjust estimates depends on having validation data tailored to each setting when exchangeability between EHRs is not assured, and the aggregation of these data almost always results in the absence of an EHR indicator.

A similar argument can be made for possible sampling error and selection bias. Predictors of patient engagement and retention in care may be dependent on a differing set of local factors for each healthcare setting, as has been observed in HIV care.^22^ Even representativeness of aggregated EHR data compared against a known population does not assure internal or external validity. For example, Epic Cosmos claims representativeness to US Census based on five data points: age, race, Hispanic ethnicity, insurance payor, and community social vulnerability.^23^ This does not assure representativeness of health measures because the Census lacks many such measures. Alternatively, if the aggregated EHR data included all healthcare providers in a region, and the study was specific to that region, there may be greater assurance of representativeness. Yet the challenge will remain if the target population is the community at-large and excludes individuals who do not or cannot access healthcare, which is a challenge even an institutional EHR study cannot overcome without community sampling.

Potential non-representativeness may manifest as selection bias in etiologic research from Epic Cosmos if the exposure and outcome are unrelated to these five data points (and differentially selected upon) and/or lack of generalizability or transportability if there are effect modifiers unrelated to these factors. While a marginal structural model using inverse probability of selection weighting may be one approach for dealing with selection bias or lack of external validity,^24,25^ a parsimonious catchment model may not be possible if the factors related to catchment differ across EHRs and an EHR indicator is not available.

### Statistical (over)Power

The largest healthcare systems in the USA have institutional EHRs that cover millions of unique patients, such as Kaiser Permanente and the Veterans Health Administration (around ten million unique patients each).^26,27^ However, aggregated platforms exceed these numbers many fold. TriNetX, as a platform that relies on de-identification as opposed to consent to include patients, surpasses 300,000,000 patients from over 120 participating healthcare organizations globally.^9^ Epic Cosmos, which is limited to participating Epic customers, includes data from close to 300,000,000 patients from approximately 1700 hospitals and 40,000 clinics.^23^ The sheer volume of data is staggering: there are over 70,000,000,000 clinical observations that may be accessed for research in TriNetX, depending on the licensing tier and research networks chosen, though some of these data may be duplicate observations.^3^ Such large datasets are also common to aggregated claims databases. For example, Merative (formerly IBM) MarketScan Database contains 273,000,000 unique patients,^28^ Optum Claims Data covers 84,000,000 patients,^29^ and the Centers for Medicare & Medicaid services claims data covers approximately 140,000,000 enrollees.^30^ With such large data, hypothesis testing will likely be accompanied by exceedingly small *p*-values. Although the importance of clinical versus statistical significance has been well-argued in the *Journal* for decades,^31,32^ it becomes even more germane when conducting an analysis on hundreds of millions of observations. Additionally, the “big data paradox” suggests that without addressing the issues in EHR data quality we are only more precisely measuring a biased effect.

## GOOD PRACTICES FOR OVERCOMING CHALLENGES TO DATA AGGREGATION

First, to help combat the “big data paradox” utilize quantitative bias analysis (QBA) anytime aggregated EHR data are used in a study. QBA provides a set of tools for estimating study effects as if systematic error did not occur.^33^ This can be done for simple “prevalence” studies with an EHR-derived health condition to more complicated associational measures of the effect of an independent variable on a dependent variable. There are even straightforward spreadsheets that can automate much of the process.^33^ As an example of QBA applied to EHR data in the presence of misclassification, see this reference, where researchers estimated prevalence of and risk factors for chronic infection with hepatitis C virus when relying on imperfectly ascertained ICD diagnostic codes.^34^

Second, purveyors of aggregated EHR might validate a sub-sample or sub-cohort of the data before and after data ETL to ensure validity of data in the resource and the underlying EHR. This will likely necessitate retrospective chart review or prospective data collection from the source healthcare system. This is feasible because many healthcare systems who participate in aggregated research platforms also have local research studies that are ongoing at the single site level that could potentially serve as validation studies. If this is not possible, others have recommended methods to corroborate findings in the aggregated EHR data, such as performing the study in duplicate by different research teams.^3^ If resources are allocated to validating data, it is imperative that this also results in updates to the aggregated platform’s CDM to accurately reflect the source EHRs data elements.

Third, retain the ability to identify the source EHR/healthcare system. This will greatly assist in the exploration for heterogeneity of measures and systematic error. For example, in the *All of Us* CDM, the source data are recorded, albeit withheld from researchers regardless of the tier of access. Even providing characteristics of the source of the data would help in establishing heterogeneity of effects. Research has shown that there are organizational domains of hospitals and clinics (e.g., structure, politics, culture, education, physical and technical infrastructure) that impact how care is provided.^35^ Without knowing more about the environment in which patients are nested, we are not able to establish nonindependence, a key assumption for most analyses, nor are we able to understand environmental drivers of heterogeneity.

Fourth, include unstructured data in the resource. This presents the distinct although not intractable additional challenge of de-identifying and anonymizing provider notes. Fortunately, there are emerging tools that can assist with this process. Stanford Medicine has developed a clinical text anonymization tool.^36^ However, if the raw unstructured data cannot be provided in these platforms, but NLP has been or could be applied to these data, purveyors must fully disclose the methods and validation of the NLP approach. In that way, researchers can vet the appropriateness of the source populating the aggregated data including benefits and limitations thereof.

The onus for the last three recommendations largely falls on the data aggregation platforms and purveyors. We recognize that these are not near-term fixes and may not even be possible depending on the ability to address privacy and confidentiality concerns. However, for users to vet the appropriateness of a proposed study when using these data sources, they must first be fully informed of the benefits and limitations of the methods used to aggregate the data, which may not always be the case. On the other hand, QBA is an “end user” task and can always be carried out with few resources by individual investigators to combat at least some of the challenges we have highlighted here.

## CONCLUSIONS

The use of aggregated EHR data is analogous to the controversial use of meta-analysis to pool study results. The recently revived, but decades-old debate condemns meta-analysis as producing a fictitious statistic considered to be the truth while struggling to capture the complexity and nuance of the studies being pooled.^37^ Common elements between pooling study results and pooling healthcare systems’ EHR data include (1) assuming they are measuring the same variables and effects, (2) assuming that any observed variation is due solely to random error, (3) excluding data that are insufficient for analysis, (4) focusing on results and not methods, and (5) conflating overly precise estimates with the correct answer. The debate argues *against* treating meta-analysis as a “default approach” to causal inference^37^; we propose the same for aggregated EHR analysis. Rather a focus on the validity of the source and extracted data is what is needed to improve the quality of research before more “big data” platforms of aggregated EHR are created.
